# Cryptococcoid Sweet syndrome or halogenoderma? Highlighting the crucial need for serum iodine testing

**DOI:** 10.1016/j.jdcr.2025.08.011

**Published:** 2025-08-20

**Authors:** Jacob R. Kilgore, Jonathan M. Joseph, Addie Walker, William Snider, Nicholas Culotta, Carson Bogatto, Aaron Coulon

**Affiliations:** a Department of Dermatology, LSU Health Sciences Center School of Medicine, New Orleans, Louisiana; b School of Medicine, Marshall University, Huntington, West Virginia; c Department of Dermatology, Ochsner, New Orleans, Louisiana

**Keywords:** contrast media, cryptococcoid Sweet syndrome, end-stage renal disease (ESRD), halogenoderma, iodine toxicity, iododerma, neutrophilic dermatosis, serum iodine levels, Sweet syndrome

## Introduction

Iododerma is a rare cutaneous reaction following exposure to iodine-containing compounds.^1^ The first reports of iododerma came from Europe in the 1850s, secondary to naturally occurring iodine salts.^1^ Halogenoderma is a broader term encompassing all halogen exposure-related dermatoses, including iododerma, bromoderma (bromine exposure), and fluoroderma (fluorine exposure). The clinical manifestations of iododerma vary widely, ranging from acute, diffuse cutaneous eruptions within hours of exposure to a more chronic onset characterized by solitary lesions.^2^ The severity varies equally, ranging from self-limited to diffuse and fatal.^2^

Due to the rarity of the disease, the underlying pathogenesis is unclear. However, end-stage renal disease (ESRD) is a predisposing factor, given that halogens are renally excreted.^3^ This case report highlights a 64-year-old male who was diagnosed with iododerma based on biopsy findings followed by confirmation with iodine levels. Given the overlapping clinical and histological findings of halogenodermas and cryptococcoid Sweet syndrome, we emphasize recognizing potential halogen exposures in similar presentations. We propose including halogen-level testing as part of the diagnostic criteria for cryptococcoid Sweet syndrome.

## Case report

A 64-year-old male with a history of ESRD on hemodialysis presented to the emergency department with fatigue, flank pain, and low hemoglobin levels. A computed tomography angiography of the abdomen showed a right flank abdominal wall hematoma, initiating interventional radiology to perform an urgent angiogram and embolization.

**Fig 1:**
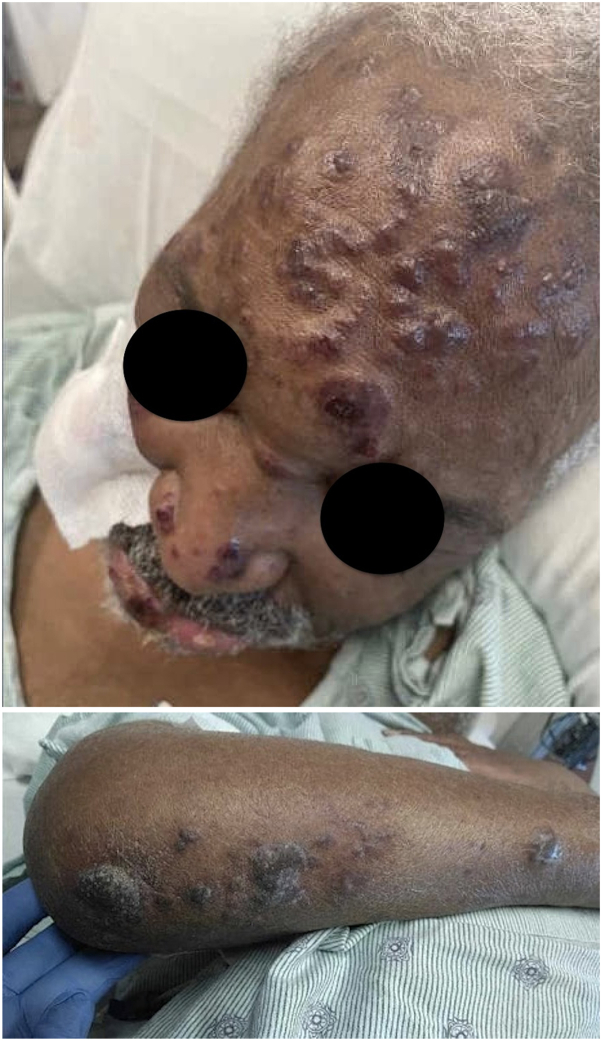
Clinical photos clinical images of the face and right forearm showing erythematous and hemorrhagic nodules and plaques.

**Fig 2:**
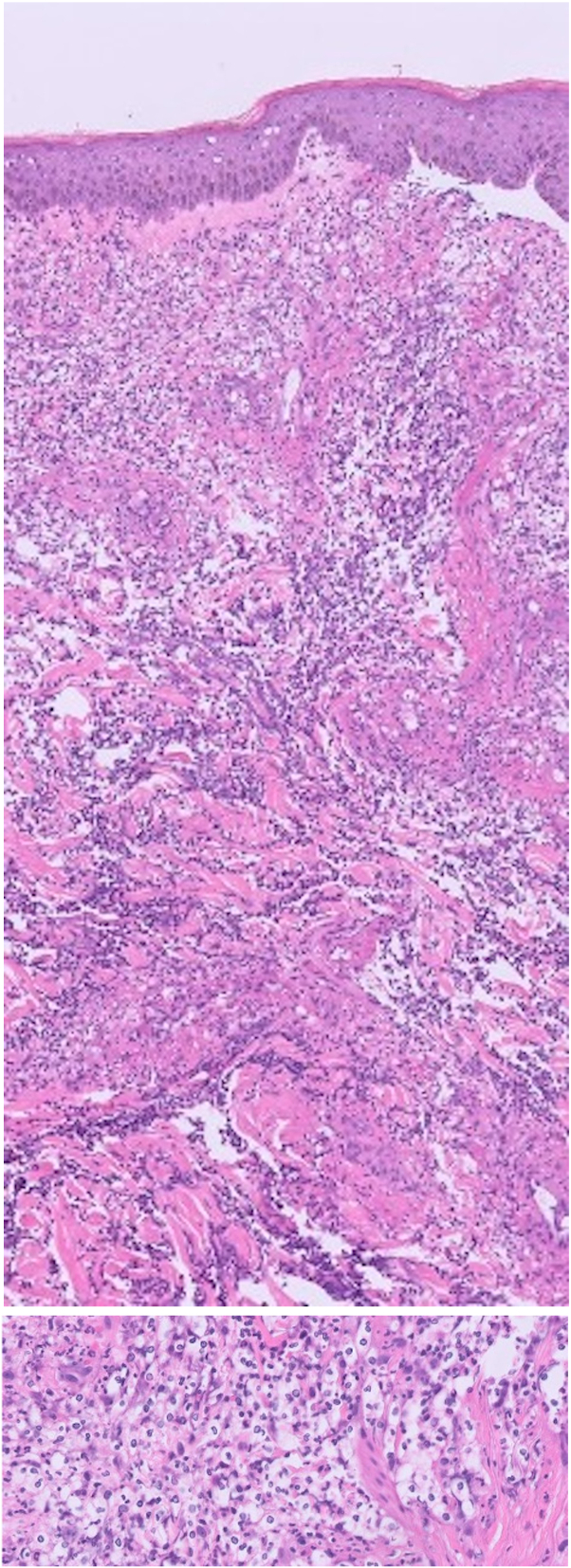
Histopathologic images punch biopsy specimen of right forearm stained with hematoxylin and eosin showing a neutrophilic dermatosis with cryptococcoid-like bodies in the dermis. Varying magnifications shown at 40× and 400×.

Thirty-six hours later, the patient developed erythematous and hemorrhagic nodules and plaques on the scalp, forehead, extremities, and hands (Fig 1). Following a punch biopsy of 2 lesions, systemic corticosteroids and broad-spectrum antibiotics were empirically started. The biopsy demonstrated a neutrophilic dermatosis with cryptococcoid-like bodies in the dermis (Fig 2). Iodine level testing revealed markedly elevated levels (>600,000 μg/L; normal range: 10-92 μg/L), confirming iododerma. Subsequently, therapeutic slow, low-efficiency dialysis was initiated, while systemic corticosteroid therapy was continued.

Despite these interventions, the patient experienced progression of his skin lesions, with sloughing of affected areas and distal extremity necrosis. Additionally, he developed new pulmonary infiltrates along with laryngeal edema and was diagnosed with pneumonia, which resulted in intubation. The patient’s condition continued to worsen, and he ultimately succumbed to the disease shortly after.

## Discussion

Iododerma is a rare cutaneous reaction to iodine-containing compounds, with severity ranging from self-limited to fatal.^2^ Iododerma falls within the broader category of halogenodermas. Various foods, medications, and products contain halogens, with iodine found in salt, dairy, egg yolk, cured meats, canned vegetables, soy, and red dye number 3 (ie, Coca-Cola). Medications containing iodine include amiodarone, iodoquinol, potassium iodide, and topical iodine-based wound care products. Additionally, iodine is present in many radiological dyes and contrast agents.^4^ Soft drinks, certain seizure medications, neostigmine, rocuronium, and other anesthesia-related drugs contain bromine.^5^ Many anesthetics and dental products contain levels of fluorine.

The pathophysiology of iododerma is currently unknown but thought to be secondary to the induction of a delayed hypersensitivity reaction via iodine acting as a hapten.^6^ Iodine itself is not immunogenic, but when bound to larger proteins, it triggers the immune response seen in the disease.^6^ In addition, since the exact pathogenesis of the vasculitis seen in iododerma remains unclear, it has been theorized that iodine increases the recruitment of polymorphonuclear leukocytes, induces apoptosis, and produces circulating immune complexes, which can result in vasculitis.^2^^,^^7^

The cutaneous manifestations of iododerma, like Sweet syndrome, can present with various morphologies, including acneiform, vegetative, hemorrhagic, bullous, and vasculitic lesions. The most common solitary presentation is a vegetative plaque that presents with a ring of peripheral pustules.^8^ When more diffuse, acneiform morphology is more common.^9^ Systemic manifestations, which are often not seen in Sweet syndrome except for fever, can include salivary gland swelling, gastrointestinal bleeding, hypotension, coma, new pulmonary infiltrations, kidney injury, and thyroid and liver abnormalities.^10^^,^^11^

Histologic findings of iododerma lesions can be variable, but typically include a diffuse mixed dermal inflammatory infiltrate with neutrophils. Intraepidermal neutrophilic microabscesses, dermal necrosis, and pale eosinophilic bodies with clear haloes may also be seen. These haloed structures resemble Cryptococcus organisms but are believed to be products of neutrophil degeneration.^8^ More chronic cases of iododerma can demonstrate similar findings in addition to pseudoepitheliomatous hyperplasia.^12^ These histologic findings are not specific to iododerma.

When creating a clinical differential for an eruption of erythematous nodules or plaque-like lesions resembling an abscess or vegetative plaque, it is important to consider diseases such as Sweet syndrome, dimorphic fungal infection, pyoderma gangrenosum, pemphigus vegetans, bullous dermatosis (particularly linear IgA bullous dermatosis), folliculitis, and even malignancy. Histologic evaluation of a biopsied lesion can narrow this differential. When pale eosinophilic bodes with clear haloes are seen within a diffuse neutrophilic infiltrate, the histologic differential diagnosis includes Cryptococcosis and cryptococcoid Sweet syndrome, as well as iododerma. While negative special stains for Gomori-Methenamine silver, Periodic Acid-Schiff, and mucicarmine readily exclude Cryptococcosis, iododerma, and cryptococcoid Sweet syndrome cannot be distinguished by histology alone.

Distinction is important as management is different. The treatment for iododerma is immediate withdrawal and discontinuation of any products that may contain iodine, including contrast from radiologic studies. Supportive care, potentially even intubation, is critical. Systemic corticosteroids, cyclosporine, and hemodialysis to remove the renally excreted halogens have been proven efficacious.^13^ The treatment for Sweet syndrome includes corticosteroids, dapsone, and colchicine; management includes looking for underlying causes through cancer screenings, complete blood counts, imaging, and autoimmune antibody levels. The current recommended workup and lab evaluation does not include halogen levels.

Given the similarities in clinical and histologic presentation between iododerma and the histologic variant of cryptococcoid Sweet syndrome and the differences in their treatment and prognosis, through a PubMed search, we reviewed previously reported cases of cryptococcoid Sweet syndrome that did not assess halogen levels (Table I). Interestingly, half of the cases listed documented a history of renal disease in addition to exposure to iodine through radiological procedures. Outcomes may have changed if these patients had iododerma and iododerma-directed treatment instead of treatment directed at Sweet syndrome.

**Table I:** Cryptococcoid Sweet syndrome literature review

Pt #	Year	Title	Pathology report	Case report	Halogen exposure
1	2013	Morphologic mimickers of Cryptococcus occurring within inflammatory infiltrates.^14^	Neutrophilic dermatosis with cryptococcosis-like bodies	A 66-year-old female was diagnosed with pneumonia and treated with antibiotics. She subsequently developed painful edematous pink papules and plaques, with biopsy confirming Sweet's syndrome, which improved following prednisone taper	Cannot be determined based on case report details
2	2017	Cryptococcoid Sweet's syndrome: 2 reports mimicking cutaneous cryptococcosis^15^	Neutrophilic infiltrate mimicking cryptococcal infection	This case report describes a rare instance of Sweet Syndrome presenting as painful, erythematous plaques and nodules in a 37-year-old woman with recurrent cervical cancer. A skin biopsy confirmed the diagnosis, and treatment with high-potency topical corticosteroids resolved the condition within 4 wk.	No
3	2019	Cryptococcoid Sweet syndrome: a clinical and histologic imitator.^16^	Pseudocryptococcal structures with neutrophilic infiltrate	18-year-old female with ANCA+ vasculitis and end-stage renal disease. After undergoing MRI presented with molluscum-like lesion on the face and blisters erythematous lesions on the backs of the hands, fever and neutrophilia	Yes. Acute kidney failure + MRI
4	2022	Sweet syndrome imitating cutaneous cryptococcal disease.^17^	Neutrophilic dermatosis with pseudocryptococcal bodies	Stage 3 CKD patient with >20 edematous round plaques and nodules	Cannot be determined based on case report details
5	2024	Cryptococcoid Sweet syndrome: a case report.^18^	Neutrophilic dermatosis with fungal-like structures	A 57-year-old man with a history of pauci-immune p-ANCA-associated crescentic glomerulonephritis on peritoneal dialysis presented with worsening anemia, melena, and later developed disseminated hemorrhagic skin lesions during hospitalization. Skin biopsies showed features suggestive of fungal organisms, but extensive testing was negative, leading to a diagnosis of cryptococcoid Sweet syndrome. He was treated with high-dose corticosteroids, resulting in significant improvement of skin lesions without scarring.	Yes. P-ANCA glomerulonephritis + developed pneumonia (assuming scans) + started developing signs
6	2014	Histiocytoid sweet syndrome with haloed myeloid cells masquerading as a cryptococcal infection^19^	Dense dermal infiltrate composed of mature neutrophils and numerous yeast-like mononuclear cells with a surrounding halo, suspicious for cryptococcal organisms	75-year-old man in which histopathologic examination showed a dense dermal infiltrate composed of mature neutrophils and numerous yeast-like mononuclear cells with a surrounding halo, suspicious for cryptococcal organisms	No
7	2020	Bullous hemorrhagic Sweet syndrome with cryptococcoid neutrophils in patients positive for antineutrophil cytoplasmic antibody without primary vasculitis^20^	Dense neutrophilic infiltrate in the mid-dermal layer with papillary dermal edema	A 70-year-old woman with end-stage renal disease, diabetes, and COPD presented with fever, abdominal pain, and respiratory distress, progressing to septic shock and hemorrhagic bullae involving her face and arms. She was diagnosed with subcutaneous infection, bilateral pneumonia, and atypical Sweet syndrome (SS) based on skin biopsy and elevated p-ANCA titers, and treated with dapsone and a prednisone taper, leading to resolution of her lesions.	Yes, ESRD + multiple CT scans + exposure to multiple antibiotics
8	2020	Bullous hemorrhagic Sweet syndrome with cryptococcoid neutrophils in patients positive for antineutrophil cytoplasmic antibody without primary vasculitis^20^	Dense neutrophilic infiltrate in the mid-dermal layer with papillary dermal edema	A 68-year-old woman with chronic anemia and hypertension developed a retropharyngeal abscess and respiratory distress, alongside hemorrhagic papules and plaques, and was diagnosed with Sweet syndrome (SS) with secondary vasculitis based on biopsy findings and p-ANCA positivity. Despite treatment with prednisone, mycophenolate mofetil, and dapsone, she succumbed to urosepsis and multiorgan failure.	Yes, pANCA, exposure to radiology exposures in ICU admission
9	2020	Bullous hemorrhagic Sweet syndrome with cryptococcoid neutrophils in patients positive for antineutrophil cytoplasmic antibody without primary vasculitis^20^	Dense neutrophilic infiltrate in the mid-dermal layer with papillary dermal edema	A 70-year-old woman with end-stage renal disease, diabetes, and recent cardiac catheterization was admitted for respiratory syncytial virus pneumonia and developed painful ulcerations on her head, upper extremities, and tongue. Examination revealed hemorrhagic granulation tissue and erythematous to violaceous exudative plaques, with a differential diagnosis including calciphylaxis, cholesterol emboli, and neutrophilic dermatoses; her condition progressed with new painful lesions despite intervention.	Yes, ESRD + pneumonia presentation to hospital
10	2014	Vancomycin-associated neutrophilic dermatitis histologically mimicking *Cryptococcus*^21^	Basophilic bodies with surrounding clear spaces occupying the upper reticular and papillary dermis	A 63-year-old man with end-stage renal disease and recurrent *Clostridium difficile* colitis developed a burning eruption on his back, arms, and thighs after starting oral vancomycin, progressing to tense bullae on his abdomen. Examination revealed erythematous papules coalescing into plaques, likely representing a drug reaction associated with vancomycin as the only recent medication change.	Yes, ESRD and on numerous medications

*CKD*, Chronic kidney disease; *COPD*, chronic obstructive pulmonary disease; *CT*, computed tomography; *ESRD*, end-stage renal disease; *ICU*, intensive care unit; *MRI*, magnetic resonance imaging.

Our case and literature review highlights the importance of surveying serum iodine levels in a patient with an acute eruption of acneiform, vegetative, hemorrhagic, bullous, or vasculitic lesions and histologic features of cryptococcoid bodies in a diffuse neutrophilic infiltrate, particularly in the setting of ESRD. Serum iodine level can be the clinicopathologic linchpin in distinguishing iododerma from the cryptococcoid histologic variant of Sweet syndrome. This distinction can be critical given the important differences in management and prognosis of the 2 conditions.

## Conclusion

Physicians should maintain a high suspicion for iododerma in patients with ESRD and a history of iodine exposure presenting with Sweet’s Syndrome-like cutaneous manifestations. Collaboration with nephrology and critical care teams is crucial, with current management for iododerma involving supportive care and corticosteroids while considering alternative immunomodulating agents and diuretics/dialysis due to renal excretion of iodine. Further research is needed to better understand the underlying pathogenesis. The diagnosis and workup of cryptococcoid Sweet Syndrome should be reevaluated and updated, with routine halogen-level assessment performed in similar cases to ensure an accurate diagnosis.

## Conflicts of interest

None disclosed.
